# Effectiveness of Columbianadin, a Bioactive Coumarin Derivative, in Perturbing Transient and Persistent *I*_Na_

**DOI:** 10.3390/ijms22020621

**Published:** 2021-01-09

**Authors:** Wei-Ting Chang, Sheng-Nan Wu

**Affiliations:** 1 Institute of Clinical Medicine, College of Medicine, National Cheng Kung University, Tainan 70101, Taiwan; cmcvecho2@gmail.com; 2 Division of Cardiovascular Medicine, Chi-Mei Medical Center, Tainan 71004, Taiwan; 3 Department of Biotechnology, Southern Taiwan University of Science and Technology, Tainan 71004, Taiwan; 4 Institute of Basic Medical Sciences, National Cheng Kung University Medical College, Tainan 70101, Taiwan; 5 Department of Physiology, National Cheng Kung University Medical College, Tainan 70101, Taiwan; 6 Department of Medical Research, China Medical University, Taichung 40402, Taiwan

**Keywords:** columbianadin, voltage-gated Na^+^ current, persistent Na^+^ current, current kinetics, hysteresis, pituitary cell, heart cell

## Abstract

Columbianadin (CBN) is a bioactive coumarin-type compound with various biological activities. However, the action of CBN on the ionic mechanism remains largely uncertain, albeit it was reported to inhibit voltage-gated Ca^2+^ current or to modulate TRP-channel activity. In this study, whole-cell patch-clamp current recordings were undertaken to explore the modifications of CBN or other related compounds on ionic currents in excitable cells (e.g., pituitary GH_3_ cells and HL-1 atrial cardiomyocytes). GH_3_-cell exposure to CBN differentially decreased peak or late component of voltage-gated Na^+^ current (*I*_Na_) with effective IC_50_ of 14.7 or 2.8 µM, respectively. The inactivation time course of *I*_Na_ activated by short depolarization became fastened in the presence of CBN with estimated *K*_D_ value of 3.15 µM. The peak *I*_Na_ diminished by 10 µM CBN was further suppressed by subsequent addition of either sesamin (10 µM), ranolazine (10 µM), or tetrodotoxin (1 µM), but it was reversed by 10 µM tefluthrin (Tef); however, further application of 10 µM nimodipine failed to alter CBN-mediated inhibition of *I*_Na_. CBN (10 µM) shifted the midpoint of inactivation curve of *I*_Na_ to the leftward direction. The CBN-mediated inhibition of peak *I*_Na_ exhibited tonic and use-dependent characteristics. Using triangular ramp pulse, the hysteresis of persistent *I*_Na_ enhanced by Tef was noticed, and the behavior was attenuated by subsequent addition of CBN. The delayed-rectifier or *erg*-mediated K^+^ current was mildly inhibited by 10 µM CBN, while it also slightly inhibited the amplitude of hyperpolarization-activated cation current. In HL-1 atrial cardiomyocytes, CBN inhibited peak *I*_Na_ and raised the inactivation rate of the current; moreover, further application of 10 µM Tef attenuated CBN-mediated decrease in *I*_Na_. Collectively, this study provides an important yet unidentified finding revealing that CBN modifies *I*_Na_ in electrically excitable cells.

## 1. Introduction

Columbianadin (CBN) is one of the main bioactive constituents isolated from the underground part of *Angelica pubescens* Maxim. f. biserrate Shan et Yuan (*Angelicae Pubescentis Radix* or “Duho [Dú huó]” in China). This compound is a coumarin-type compound (an angular dihydrofuranocoumarin), which is increasingly recognized to have various biological activities that include analgesic, anti-inflammatory, and antineoplastic effects [1,2,3,4,5,6,7,8,9,10,11,12,13]. This compound has been reported to induce changes in cellular proliferation or apoptosis [12,14]. It has been also previously demonstrated that carrageenan or lipopolysaccharide-induced inflammatory reaction was ameliorated by the application of CBN [1,2,4,7].

There is growing evidence that CBN could be a regulator of membrane ionic currents [9,10]. CBN-mediated analgesia was previously disclosed to be closely associated with its adjustments on the expression of TRPV1 channels [8,9,15]. Earlier studies have also reported the ability of this compound to inhibit the strength of voltage-gated Ca^2+^ currents in dorsal root ganglion neurons derived from mice, suggesting a possible link to its analgesic action in neuropathic pain model [10,16]. Alternatively, it has been previously revealed to inhibit depolarization-induced Ca^2+^ uptake as well to suppress prolactin release in pituitary GH_3_ cells [16,17].

There are nine isoforms (i.e., α-subunits) of voltage-gated Na^+^ (Na_V_) channel (i.e., Na_V_1.1-1.9), which are presently known to exist in mammalian tissues, including endocrine system [18,19,20]. Earlier reports have demonstrated that several inhibitors known to preferentially block the late component of voltage-gated Na^+^ current (*I*_Na_), such as ranolazine (Ran), KMUP-1, or sesamin (SSM) [21,22]. Na_V_ channels are also expressed in nonexcitable cells at a lower level [19]. However, to date, the need of how CBN or other related compounds is capable of interacting with Na_V_ channels to modify the strength and/or gating of *I*_Na_ in different types of electrically excitable cells remains largely unmet.

In light of the above-described statements, the purpose of this study was to investigate whether the presence of CBN is capable of causing any perturbations on various types of ionic currents in pituitary tumor (GH_3_) cells that include voltage-gated *I*_Na_, persistent *I*_Na_ (*I*_Na[P]_), *erg*-mediated K^+^ current (*I*_K(erg)_), delayed-rectifier K^+^ current (*I*_K(DR)_), and hyperpolarization-activated cation current (*I*_h_). GH_3_ cells have been used as a cell model to study the gating properties of *I*_Na_ [23,24], and Na_V_ channels are known to be native to all secretory pituitary cells [20]. In particular, the results of the present experiments highlight a notion that CBN can yield a depressant action on *I*_Na_ in a concentration-, time-, and state-dependent manner in excitable cells (e.g., GH_3_ cells and HL-1 cardiomyocytes). The yet unidentified depressant action of this agent on *I*_Na_ reported herein is thought to be through its interaction with the Na_V_ channel in the open or open/inactivated state.

## 2. Results

### 2.1. Effect of Columbianadin (CBN) on Voltage-Gated Na^+^ Current (I_Na_) Recorded from GH_3_ Cells

**Figure 1:**
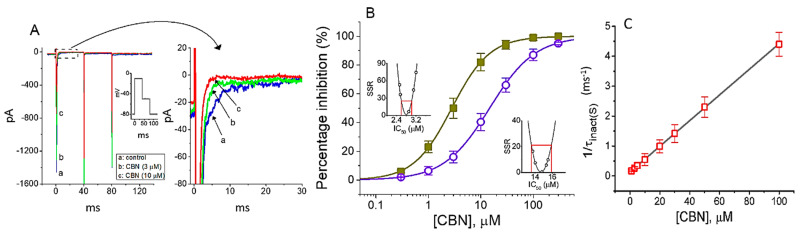
Effect of columbianadin (CBN) on voltage-gated Na^+^ current (*I*_Na_) in pituitary tumor (GH_3_) cells. The experiments were undertaken in cells bathed in Ca^2+^-free Tyrode’s solution, and the recording electrode was filled up with Cs^+^-containing solution. (**A**) Typical current traces obtained in the control (a, i.e., CBN was not present) and in the presence of 3 µM CBN (b) or 10 µM CBN (c). The insert illustrates the voltage protocol applied. The right panel indicates an expanded record from the dashed box of (**A**). (**B**) Concentration-dependent relationship of CBN on peak (○) and late (■) components of *I*_Na_ elicited by rapid membrane depolarization (mean ± SEM; *n* = 8 for each point). Insets show confidence assessment of best-fit parameter values in the peak and late component of *I*_Na_ in response to short depolarizing voltage pulse from −80 to −10 mV. Red line shown in each inset is placed at parameter value (i.e., IC_50_) at which the sum of squared residuals (SSR) in the peak or late component of *I*_Na_ amounts to 21% or 25% respectively, while the parameter range corresponds to the approximate 95% confidence interval. (**C**) The relation of 1/τ_inact(S)_ (i.e., the reciprocal of the slow component of current inactivation) versus the CBN concentration (mean ± SEM; *n* = 8 for each point). The value of *I*_Na_ inactivation was measured as the examined cells were 40 ms depolarized from −80 to −10. Forward (*k*_+1_*) or backward (*k*_−1_) rate constant, determined from the slope or the *y*-axis interacept of the interpolated line, was yielded to be 0.0428 ms^−1^µM^−1^ or 0.1348 ms^−1^, respectively.

In the first stage of the whole-cell experiments, we examined the effects of CBN on the amplitude and gating of *I*_Na_ in response to abrupt membrane depolarization. GH_3_ cells were immersed in Ca^2+^-free, Tyrode’s solution containing 10 mM TEA, and we filled up the recording electrode with a Cs^+^-containing solution. As depicted in Figure 1A, within 1 min of exposing cells to CBN at a concentration of 3 or 10 µM, the amplitude in the peak and late (or end-pulse) components of *I*_Na_ activated by rapid membrane depolarization from −80 to −10 mV was progressively decreased. For example, as short step depolarization from −80 to −10 mV with a duration of 40 ms was applied (indicated in the inset of Figure 1A) to activate *I*_Na_, the addition of 10 µM CBN caused a conceivable decrease in the peak or late amplitude of *I*_Na_ to 913 ± 63 or 5.2 ± 0.8 pA (*n* = 9, *p* < 0.05), respectively, from the control values of 1521 ± 212 or 12.1 ± 0.9 pA (*n* = 9). After removal of CBN, the peak or late amplitude of the current returned to 1456 ± 186 or 11.9 ± 0.9 pA (*n* = 8, *p* < 0.05).

Figure 1B illustrates that the addition of CBN to the bath can concentration-dependently depress the amplitude of peak or late *I*_Na_ evoked in response to rapid membrane depolarization. According to the modified Hill equation detailed under Materials and Methods, the IC_50_ value required for CBN-induced inhibition of peak or late *I*_Na_ observed in GH_3_ cells was calculated to be 14.7 or 2.8 µM, respectively, the value of which was evidently distinguishable between its effects on these two components of the current. The results, therefore, enable us to unravel that CBN has a depressant action on the peak and late *I*_Na_ natively expressed in GH_3_ cells and that this agent tends to be selective for late over peak *I*_Na_ in response to rapid depolarizing pulse.

### 2.2. Kinetic Study of CBN Action on I_Na_

It was noticed that the inhibitory effects of CBN on *I*_Na_ in GH_3_ cells could not only emerge instantaneously (i.e., they vary in time) but they also occurred in a time- and concentration-dependent manner. In other words, the interaction of the CBN molecule with Na_V_ channel would develop with time after the channels are bound with the compound. In this regard, in attempts to provide quantitative estimation of CBN-induced block of *I*_Na_, we further analyzed the time constant of the slow component of *I*_Na_ inactivation (τ_inact(S)_). The concentration-dependence of CBN-induced block for *I*_Na_ in response to abrupt membrane depolarization is illustrated in Figure 1C. It is, therefore, evident from these data that the exposure to CBN resulted in a concentration-dependent increase in the rate constant (i.e., 1/τ_inact(S)_) of the slow component in *I*_Na_ inactivation. There was a linear relationship between the 1/τ_inact (S)_ value and the CBN concentration with a correlation coefficient of 0.96 (Figure 1C). The blocking (*k*_+1_*) or unblocking (*k*_−1_) rate constant derived from such minimum binding (forward) and unbinding (backward) scheme was then calculated to be 0.0428 ms^−1^µM^−1^ or 0.1348 ms^−1^, respectively; consequently, the value of dissociation constant (*K*_D_ = *k*_−1_/*k*_+1_*) was yielded to be 3.15 µM. Moreover, this value achieved by this reaction scheme was found to be nearly similar to the IC_50_ value required for CBN-mediated inhibition of the late *I*_Na_ measured at the end of depolarizing voltage pulse; however, it tends to be higher than that for block of peak *I*_Na_ (Figure 1B).

### 2.3. Comparison among Effects of CBN, CBN Plus Sesamin (SSM), CBN Plus Ranolazine (Ran), CBN Plus Tetrodotoxin (TTX), CBN Plus Nimodipine (Nim), and CBN Plus Tefluthrin (Tef) on Peak I_Na_ Recorded from GH_3_ Cells

**Figure 2:**
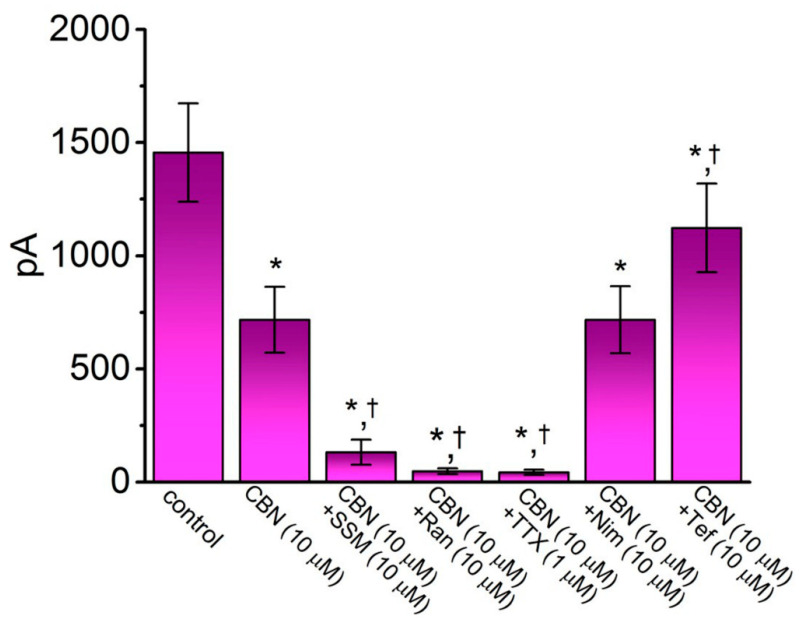
Comparison among effects of CBN, CBN plus sesamin (SSM), CBN plus ranolazine (Ran), CBN plus tetrodotoxin (TTX), CBN plus nimodipine (Nim), and CBN plus tefluthrin (Tef) on the amplitude of peak *I*_Na_ observed in GH_3_ cells. Cells were bathed in Ca^2+^-free, Tyrode’s solution, and the recording pipet was filled up with Cs^+^-containing solution. The *I*_Na_ was activated by 50-ms depolarizing voltage command from −80 to −10 mV, and current amplitude at the beginning of the voltage pulse was measured. Each bar represents the mean ± SEM (*n* = 7–8). * indicates significantly different from control (i.e., none of the agents were present) (*p* < 0.05), and † indicates significantly different from CBN (10 µM) alone group (*p* < 0.05).

In another set of whole-cell experiments, we tested the possible effects of CBN, CBN plus different compounds, including SSM, Ran, TTX, Nim, and Tef, on the peak amplitude of *I*_Na_ activated in response to rapid membrane depolarization from −80 to −10 mV. Either SSM, Ran, or TTX has been previously reported to inhibit *I*_Na_ effectively [21,25], Nim is a specific inhibitor of L-type Ca^2+^ current [26], whereas Tef is a type-I pyrethroid insecticide known to activate *I*_Na_ [27]. As summarized in Figure 2, CBN (10 µM) produced an inhibitory effect on the peak amplitude of *I*_Na_. In continued presence of CBN (10 µM), the further addition of either SSM (10 µM), Ran (10 µM), or TTX (1 µM) was effective at decreasing *I*_Na_ amplitude further. However, as cells were continually exposed to CBN (10 µM), subsequent application of Tef (10 µM), but not Nim (10 µM), was able to attenuate CBN-induced block of peak *I*_Na_ seen in GH_3_ cells.

### 2.4. Lack of Effect of CBN on the Steady-State Activation Curve of I_Na_

**Figure 3:**
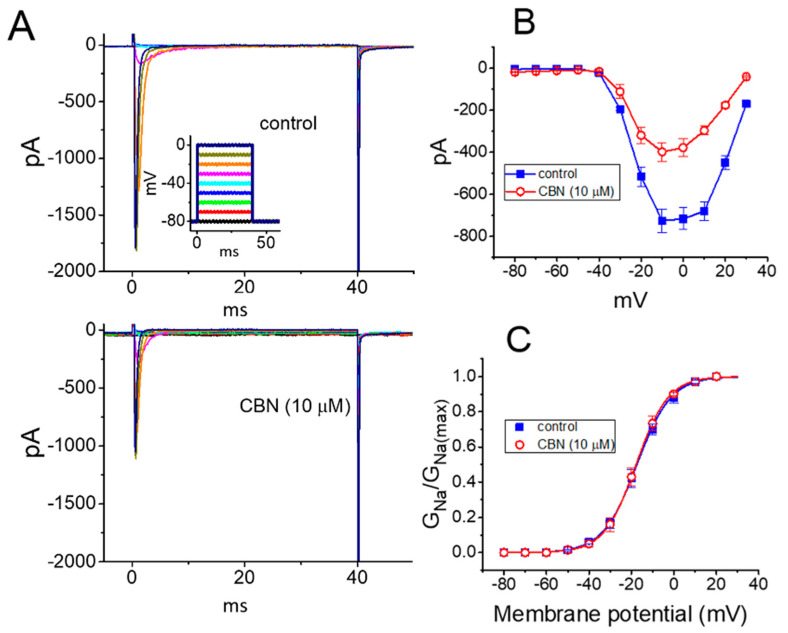
Effect of CBN on the current versus voltage (*I-V*) and conductance versus voltage relationship of *I*_Na_ identified in GH_3_ cells. (**A**) Representative current traces activated by a series of voltage commands ranging from −80 to 0 mV in 10-mV increments. Current traces in the upper part are control, while those in the lower part were obtained in the presence of 10 µM CBN. Inset in the upper part shows the voltage-clamp profile applied. (**B**) Mean *I-V* relationship of peak *I*_Na_ obtained in the absence (■) and presence (○) of 10 µM CBN (mean ± SEM; *n* = 7 for each point). Current amplitude was measured at the beginning of each voltage command. (**C**) Mean conductance versus voltage relationship of peak *I*_Na_ taken in the control (■) and during the exposure (○) to 10 µM CBN (mean ± SEM; *n* = 7 for each point). Smooth line indicates the goodness-of-fit to the modified Boltzmann equation. Of notice, no obvious difference in conductance versus potential relationship with or without CBN addition was demonstrated.

To characterize the inhibitory effect of CBN on *I*_Na_, we next studied whether this compound might yield any changes in the steady-state action curve of *I*_Na_ recorded from GH_3_ cells. Figure 3A depicts representative *I*_Na_ traces activated by a series of voltage pulses ranging between −80 and 0 mV in 10-mV increments from a holding potential of −80 mV. The mean *I-V* relationship of peak *I*_Na_ with or without the application of 10 µM CBN is illustrated in Figure 3B. It was noted that the value of threshold or maximal voltage for activation of *I*_Na_ did not differ between the absence and presence of 10 µM CBN. Figure 3C depicts the relationship of *I*_Na_-conductance (*G*_Na_) versus membrane potential collected in the control (i.e., CBN was not present) and during exposure to 10 µM CBN. In the control, *V*_1/2_ = −17.4 ± 2.2 mV and *q* = 3.1 ± 0.8 *e* (*n* = 7), while in the presence of 10 µM CBN, *V*_1/2_ = −17.8 ± 2.3 mV and *q* = 3.2 ± 0.9 *e* (*n* = 7). Certainly, the experimental results reflect that the value of neither *V*_1/2_ nor the apparent gating charge (*q*) for the steady-state activation curve of *I*_Na_ is perturbed in the presence of 10 µM CBN.

### 2.5. Steady-State Inactivation Curve of Peak I_Na_ Obtained in the Absence or Presence of CBN

**Figure 4:**
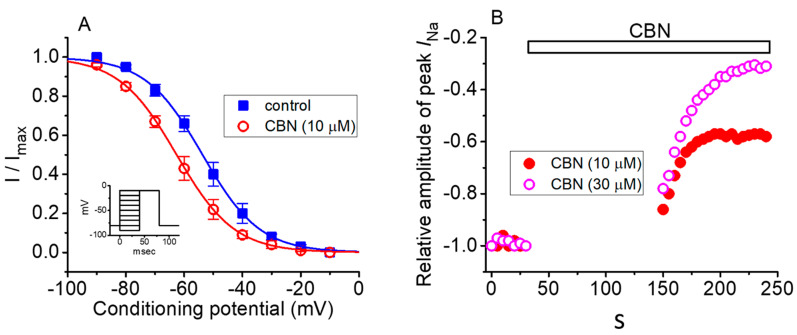
CBN-mediated change in the steady-state inactivation curve of peak *I*_Na_ (**A**) and its tonic- and use-dependent block of peak *I*_Na_ (**B**) in GH_3_ cells. In these experiments (**A**), cells were immersed in Ca^2+^-free Tyrode solution, while the examined cells were held at −80 mV, and a series of the voltage pulses ranging between −90 and −10 mV (as indicated in inset) were given. ■: control (i.e., CBN was not present) and ○: in the presence of 10 µM CBN. Each point represents the mean ± SEM (*n* = 8). The sigmoid line shows the goodness-of-fit to the modified Boltzmann equation. Note that the inactivation curve of *I*_Na_ was shifted to a negative direction in the presence of 10 µM CBN; however, there was lack of change in the apparent gating charge of the current. (**B**) Tonic- and use-dependent block of peak *I*_Na_ by CBN. The cell was held at −80 mV and the depolarizing pulse from −80 to −10 mV (40 ms in duration) was applied at 0.2 Hz. The application of CBN (10 or 30 µM) is illustrated by a horizontal bar. Changes in the relative amplitude of peak *I*_Na_ during exposure to 10 or 30 µM CBN are illustrated. The peak *I*_Na_ in the control was taken as -1.0. Immediately after the voltage pulses were stopped, various concentrations (10 or 30 µM) of CBN were added to the bath. The repetitive depolarizing pulses to −10 mV at 0.2 Hz were applied again 2 min after the cessation of command pulses, but still in the continued presence of CBN (10 µM [●] or 30 µM [○]). Of notice, in the presence of CBN, the peak *I*_Na_ activated by the first depolarizing step following a long pause (around 2 min) had been already suppressed (i.e., tonic inhibition), and, during the repetitive stimuli, the amplitude of peak *I*_Na_ was further reduced exponentially (i.e., use-dependent inhibition).

Next, we assessed the steady-state inactivation curve of *I*_Na_ taken with or without the CBN addition. Two-step voltage-clamp protocol was used in this set of whole-cell experiments. The data with respect to the relationship of conditioning potential versus normalized amplitude of peak *I*_Na_ were constructed and a measure of goodness-of-fit for a modified Boltzmann equation fitted to the data set was thereafter performed. As depicted in Figure 4, the steady-state inactivation curve of *I*_Na_ could be modified in the presence of CBN (10 µM). In the control, the resultant value for *V*_1/2_ or *q* was estimated to be −53.7 ± 2.5 mV and 2.6 ± 0.9 *e* (*n* = 8), respectively; however, during GH_3_-cell exposure to 10 µM CBN, that of *V*_1/2_ or *q* was −62.6 ± 2.6 mV or 2.5 ± 0.9 *e* (*n* = 8), respectively. The data therefore enable us to reflect that the midpoint for the steady-state inactivation curve of peak *I*_Na_ was shifted along the voltage axis in a leftward direction by about 9 mV; however, there was void of clear adjustments in the apparent gating charge of the curve.

### 2.6. Use-Dependence of CBN-induced Inhibition of Peak I_Na_

This set of experiments was undertaken to evaluate the use-dependence property of CBN-mediated inhibition of peak *I*_Na_. As shown in Figure 4B, the depolarizing pulses from −80 to −10 mV (40 ms in duration) were applied at 0.2 Hz. Under control condition (i.e., CBN was not present), no decline in the peak *I*_Na_ amplitude elicited at this simulation protocol was observed for more than 5 min. When peak *I*_Na_ remained constant in Ca^2+^-free, Tyrode’s solution, the depolarizing pulses were stopped, and CBN (10 or 30 µM) was then applied to the bath. After 2 min of cessation, the depolarizing pulses from −80 to −10 mV at 0.2 Hz were given to the cells again. The relative amplitude of peak *I*_Na_ with respect to that before the addition of CBN was constructed. It is illustrated in Figure 4B. Of notice, CBN-mediated inhibition of peak *I*_Na_ consists of both tonic and use-dependent components. For example, in the presence of 10 µM CBN, after a 2-min pause, the peak *I*_Na_ evoked by the first voltage step was suppressed by 14% ± 4% (*n* = 7) (i.e., tonic inhibition). Moreover, following the repetitive stimuli, peak *I*_Na_ amplitude was further reduced to a constant level in an exponential fashion (i.e., use-dependent inhibition). The percentage inhibition of peak *I*_Na_ following the repetitive stimuli was increased to 39% ± 5% (*n* = 7). As the CBN concentration was increased to 30 mM, the peak *I*_Na_ amplitude at the first depolarizing pulse and during subsequent repetitive stimuli were significantly suppressed by 18% ± 4% and 72% ± 5% (*n* = 7), respectively. The results indicated that CBN-mediated block of peak *I*_Na_ is composed of two components, namely, tonic and use-dependent components.

### 2.7. The Enhanced Amplitude by Tef of Persistent Na^+^ Current (I_Na(P)_) Attenuated by CBN

**Figure 5:**
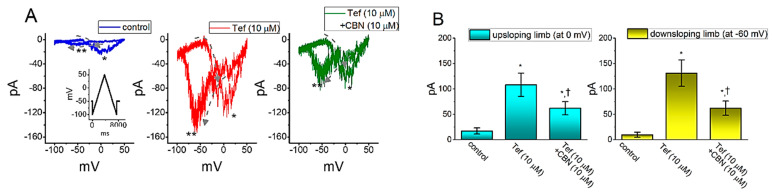
Inhibitory effect of CBN on tefluthrin (Tef) mediated increase in persistent *I*_Na_ current (*I*_Na(P)_) in GH_3_ cells. In these whole-cell current recordings, the cell was voltage-clamped at −50 mV and the isosceles-triangular ramp voltage with a duration of 7.4 s (i.e., ramp speed = ±0.04 mV/ms) was applied to activate *I*_Na(P)_ in response to the upsloping (from −100 to +50 mV) and downsloping (from +50 to −100 mV) ramp voltage-clamp command. (**A**) Representative current trace obtained in the control (left) and during the exposure to 10 µM tefluthrin (Tef) (middle) or to 10 µM Tef plus 10 µM CBN. Inset in the left panel is the voltage-clamp protocol applied for all panels in (**A**), and dashed arrows in each panel show the direction of *I*_Na(P)_ in which time passes during the elicitation by the voltage-clamp command of upright isosceles-triangular ramp with a duration of 7.4 s. * and ** indicate the amplitude of *I*_Na(P)_ activated by the upsloping (or forward) limb and downsloping (or backward) limb of the long-lasting triangular ramp pulse, respectively. (**B**) Summary bar graph showing the effects of tefluthrin (Tef, 10 µM) and Tef (10 µM) plus CBN (10 µM) on *I*_Na(P)_ amplitude activated by the upsloping and downsloping limb of 7.4 s triangular ramp pulse. Current amplitude shown in the left side was taken at the level of 0 mV in the situations where the upsloping limb of triangular pulse was applied to evoke *I*_Na(P)_, while that in the right side was at the level of −60 mV during the downsloping end of the pulse. * indicates significantly different from control (*p* < 0.01), and † indicates significantly different Tef (10 µM) alone group (*p* < 0.05).

We next continued to explore whether the presence of CBN could modify the Tef-enhanced *I*_Na(P)_ elicited in response to the slow triangular ramp pulse in GH_3_ cells. In these experiments, as the whole-cell current recordings were firmly established, we held the examined cell in voltage clamp at −50 mV and a 7.4 s upright isosceles-triangular ramp pulse ranging between −100 and +50 mV with a ramp speed of ±0.04 mV/ms was applied. Of notice, in keeping with previous observations [27,28], when the cells were exposed to 10 µM Tef alone, the amplitude of *I*_Na(P)_ activated at both upsloping (forward) and downsloping (backward) ends of such triangular ramp voltage was remarkably raised (Figure 5). For example, as cells were exposed to 10 µM Tef, the peak *I*_Na(P)_ amplitude measured at the level of 0 mV under the rising phase of triangular ramp pulse was considerably raised to 108 ± 23 pA (*n* = 8, *p* < 0.01) from a control value (measured at the same level of membrane potential) of 17 ± 6 pA (*n* = 8). Meanwhile, in the presence of 10 µM Tef, the peak *I*_Na(P)_ amplitude measured at −60 mV during the declining phase of triangular ramp was concurrently raised from 10 ± 5 to 131 ± 26 (*n* = 8, *p* < 0.01). Moreover, as shown in Figure 5, when cells were continually exposed to 10 µM Tef, the subsequent addition of 10 µM CBN further caused a significant reduction in the peak *I*_Na_ amplitude measured either at 0 mV during the upsloping end of the triangular ramp pulse or at −60 mV during the downsloping end to 62 ± 13 or 62 ± 14 pA (*n* = 8, *p* < 0.05), respectively. These observations, therefore, prompted to reflect that the voltage-dependent hysteresis of *I*_Na(P)_ markedly occurred in the presence of Tef and that the subsequent application of CBN was capable of attenuating Tef-induced increase in the amplitude of *I*_Na(P)_ activated by either forward or backward end of long-lasting triangular ramp pulse observed in GH_3_ cells.

### 2.8. Effect of CBN on erg-Mediated K^+^ Current (I_K(erg)_) in GH_3_ Cells

**Figure 6:**
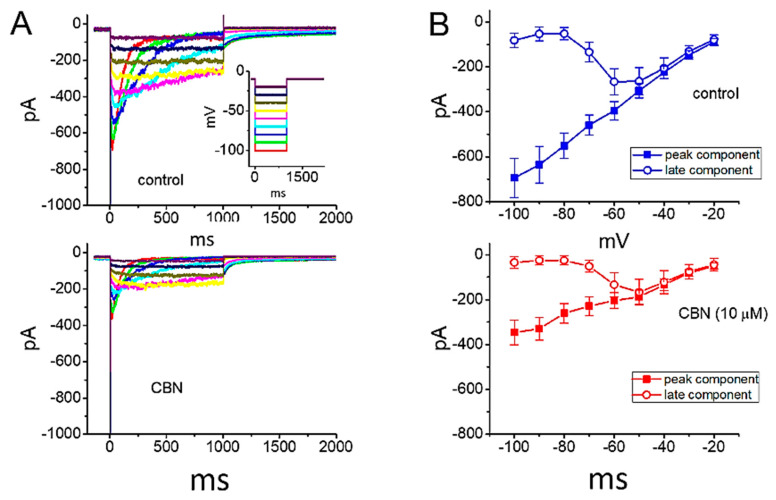
Inhibitory effect of CBN on *erg*-mediated K^+^ current (*I*_K(erg)_) identified in GH_3_ cells. In these experiments, we immersed cells in high-K^+^, Ca^2+^-free solution, and we filled up the pipet using K^+^-containing solution. (**A**) Representative current traces obtained in the absence (upper) and presence (lower) of 10 µM CBN. Inset in the upper panel shows the voltage-clamp protocol applied. (**B**) Mean *I-V* relationship of peak *I*_K(erg)_ (filled squares) and late *I*_K(erg)_ (open circles) recorded from these cells (mean ± SEM; *n* = 8 for each point). The upper or lower panel depicts the *I-V* relationship of the current in the absence (blue color) or presence (red color) of 10 µM CBN, respectively. Current amplitude (peak or late component) was measured at the start or the end of 1-s hyperpolarizing pulse from −10 mV to various voltage steps ranging between −100 and −20 mV in 10-mV step.

We also extended to evaluate whether CBN could modify voltage-gated K^+^ current (e.g., *I*_K(erg)_) enriched in GH_3_ cells. In order to amplify the magnitude of *I*_K(erg)_, we bathed cells in high-K^+^, Ca^2+^-free solution, and the recording electrode was backfilled with K^+^-containing solution. The composition of these solutions used is detailed under Materials and Methods. In this set of whole-cell experiments, in order to evoke *I*_K(erg)_, the examined cell was voltage-clamped at −10 mV and a series of 1 s hyperpolarizing pulses ranging between −100 and −20 mV was thereafter applied. As illustrated in Figure 6, the application of 10 µM CBN was noticed to decrease the amplitude of deactivating *I*_K(erg)_ observed throughout the entire voltage-clamp range examined. For example, as cells were exposed to 10 µM CBN, the peak or late amplitude of *I*_K(erg)_ taken at the level of −100 mV was decreased to 346 ± 55 or 34 ± 26 pA (*n* = 8, *p* < 0.05) from a control value of 694 ± 87 or 81 ± 32 pA, respectively. After washout of the agent, current amplitude at the beginning or end of hyperpolarizing pulse from −10 to −100 mV returned to 654 ± 85 or 78 ± 29 pA, respectively (*n* = 7, *p* < 0.05). These data indicate that CBN at a concentration of 10 µM can depress the amplitude of hyperpolarization-elicited *I*_K(erg)_ by around 50% in GH_3_ cells.

### 2.9. Effect of CBN on Delayed-Rectifier K^+^ Current (I_K(DR)_) in GH_3_ Cells

**Figure 7:**
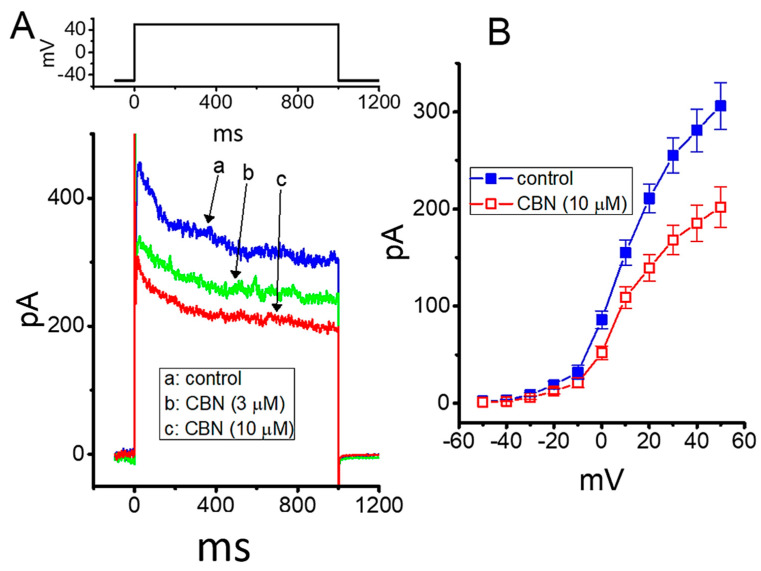
Effect of CBN on delayed-rectifier K^+^ current (*I*_K(DR)_) in GH_3_ cells. In this set of experiments, cells were bathed in Ca^2+^-free, Tyrode’s solution containing 1 µM TTX, and we filled the electrode with K^+^-containing solution. (**A**) Representative current traces obtained in the absence (a) and presence of 3 µM CBN (b) or 10 µM CBN (c). (**B**) Mean *I-V* relationship of *I*_K(DR)_ without (■) or with (○) the addition of 10 µM CBN (mean ± SEM; *n* = 7 for each point). Current amplitude was taken at the end of each depolarizing voltage command from a holding potential of −50 mV with a duration of 1 s.

In the next series of experiments, we continued to test whether the presence of CBN might modify another type of K^+^ current (i.e., *I*_K(DR)_) enriched in these cells. Cells were bathed in Ca^2+^-free, Tyrode’s solution, which contained 1 µM TTX, while the recording pipette used was filled with K^+^-containing solution. As illustrated in Figure 7A,B, CBN at a concentration of 3 or 10 µM could mildly suppress the amplitude of *I*_K(DR)_ activated by membrane depolarization; however, the inactivation time course of the current was little altered. For example, as cells were exposed to 10 µM CBN, the *I*_K(DR)_ activated by depolarizing pulse from −50 to +50 mV was decreased by 34% ± 2% to 202 ± 21 pA (*n* = 8, *p* < 0.05) from a control value of 306 ± 24 pA (*n* = 8). After the compound was washed out, current amplitude returned to 294 ± 23 pA (*n* = 7). Figure 7B depicts the mean *I-V* relationship of *I*_K(DR)_ obtained in the absence or presence of 10 µM CBN.

### 2.10. Effect of CBN on Hyperpolarization-Activated Cation Current (I_h_) in GH_3_ Cells

**Figure 8:**
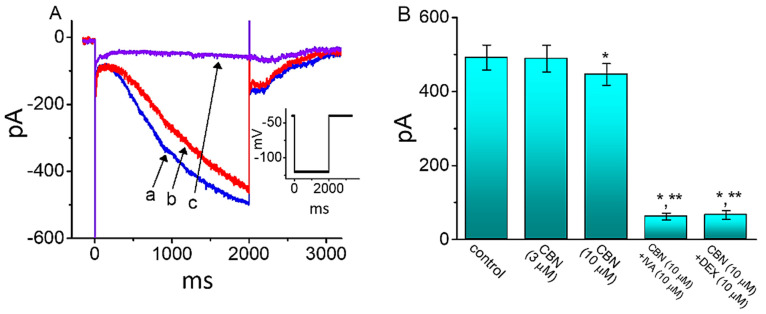
Effect of CBN on hyperpolarization-activated cation current (*I*_h_) in GH_3_ cells. Cells were bathed in Ca^2+^-free, Tyrode’s solution and the pipet were backfilled with K^+^-containing solution. (**A**) Representative current traces obtained in the absence (a) and presence of 10 µM CBN (b) or 10 µM CBN plus 10 µM ivabradine (IVA) (c). Inset depicts the voltage protocol used. (**B**) Summary bar graph demonstrating effect of 10 µM CBN and 10 µM CBN plus 10 µM ivabradine (IVA) on the amplitude of *I*_h_ (mean ± SEM; *n* = 7 for each bar). Current amplitude was taken at the end of 2 s hyperpolarizing pulse from −40 to −120 mV. * indicates significantly different from control (*p* < 0.05) and ** indicates significantly different from 10 µM CBN alone group (*p* < 0.05).

We next investigated how cell exposure to CBN could perturb the amplitude and gating of *I*_h_ identified in GH_3_ cells [29]. In these experiments, we bathed cells in Ca^2+^-free, Tyrode’s solution containing 1 µM TTX and the recording electrode was filled up with K^+^-containing solution. As demonstrated in Figure 8, the application of 10 µM CBN to the bath mildly lessened *I*_h_ amplitude activated by 2 s step hyperpolarization from −40 to −120 mV, which was noticed to coincide with a lengthening in the activation time constant of *I*_h_. However, no change in the instantaneous amplitude of *I*_h_ as recently reported [30] was detected in the presence of CBN. For example, when cells were exposed to 10 µM CBN, the *I*_h_ amplitude measured at the end of 2-s hyperpolarizing pulse was mildly but significantly reduced by 9% ± 1% to 446 ± 29 pA (*n* = 7, *p* < 0.05) from a control value of 492 ± 34 pA. Moreover, subsequent application of 10 µM ivabradine (IVA) or 10 µM dexmedetomidine (DEX), but still in the presence of CBN, was able to decrease *I*_h_ magnitude further, as demonstrated by a significant reduction in current amplitude to 62 ± 9 pA (*n* = 7, *p* < 0.05) or 66 ± 12 pA (*n* = 7, *p* < 0.05). IVA or DEX was previously reported to block *I*_h_ effectively [15,31]. It is evident, therefore, that cell exposure to CBN can result in a mild decrease in IVA-sensitive *I*_h_ amplitude activated in response to long-lasting membrane hyperpolarization.

### 2.11. Inhibitory Effect of CBN on I_Na_ Identified from HL-1 Cardiomyocytes

**Figure 9:**
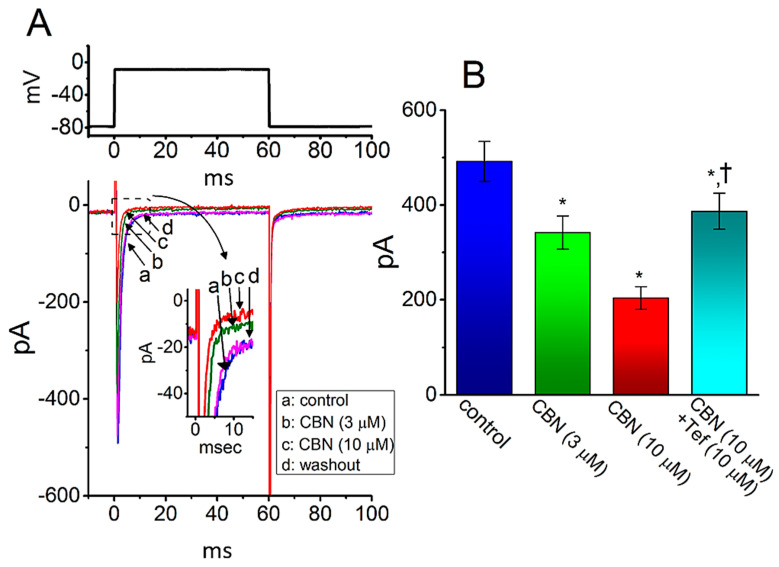
Effect of CBN on the amplitude of *I*_Na_ recorded from HL-1 atrial cardiomyocytes. The whole-cell current recordings were conducted in cells, which were bathed in Ca^2+^-free, Tyrode’s solution, and we backfilled the electrode with Cs^+^-containing solution. (**A**) Representative current traces activated by 60-ms depolarizing pulse from −80 to −10 mV (indicated in the upper part). a: control (i.e., CBN was not present), b: 3 µM CBN, c: 10 µM CBN, and d: washout of the agent. Inset in (**A**) indicates the expanded record from dashed box. (**B**) Summary bar graph demonstrating effect of CBN and CBN plus tefluthrin (Tef) on the peak amplitude of *I*_Na_ in HL-1 cells (mean ± SEM; *n* = 8 for each bar). Current amplitude was taken at the start of depolarizing pulse from −80 to −10 mV. * indicates significantly different from control (*p* < 0.05) and † indicates significantly different from CBN (10 µM) alone group (*p* < 0.05).

The CBN-perturbed changes in the properties of *I*_Na_ seen in GH_3_ cells may be different from those in heart cells. As such, in a final set of experiments, we attempted to explore whether the *I*_Na_ existing in another type of excitable cells (e.g., HL-1 atrial cardiomyocytes) can be influenced by the presence of CBN. For these current recordings, HL-1 cells were bathed in Ca^2+^-free, Tyrode’s solution containing 10 mM TEA and the recording electrode was filled with Cs^+^-containing solution. As illustrated in Figure 9, as the examined cell was depolarized from −80 to −10 mV, the *I*_Na_ with rapid activation and inactivation was readily elicited [27]. As cells were exposed to 3 or 10 µM CBN, the peak amplitude of *I*_Na_ was progressively decreased, which coincided with an increase in inactivation rate constant of the current activated in response to rapid membrane depolarization. For example, in the presence of 10 µM CBN, the peak *I*_Na_ was decreased from 492 ± 42 to 204 ± 24 pA (*n* = 8, *p* < 0.05). Concomitantly, the τ_inact(S)_ value of *I*_Na_ inactivation was also reduced from 6.2 ± 0.9 to 3.1 ms (*n* = 8, *p* < 0.05). After washout of the agent, current amplitude returned to 486 ± 38 pA (*n* = 8, *p* < 0.05). Moreover, as cells were continually exposed to 10 µM CBN, the subsequent addition of 10 µM Tef was able to reverse CBN-mediated inhibition of peak *I*_Na_ effectively in HL-1 cells. Therefore, the emerging data showed that, similar to *I*_Na_ in GH_3_ cells described above, the *I*_Na_ in HL-1 cells was subjected to clear inhibition by CBN, although the results are to some extent distinguishable from those obtained from dorsal root ganglion neurons [10].

## 3. Discussion

The principal results of the present work are that (a) the presence of CBN could depress *I*_Na_ in a concentration-, time-, and state-dependent manner in GH_3_ cells, (b) this compound differentially inhibited the peak and late amplitudes of *I*_Na_ activated by rapid membrane depolarization with effective IC_50_ of 14.7 and 2.8 µM, respectively, (c) it shifted the midpoint of *I*_Na_ inactivation curve along the voltage axis to a negative potential, despite inability to alter the activation curve of the current, (d) it suppressed peak *I*_Na_ in a tonic and use-dependent manner, (e) subsequent addition of CBN was capable of depressing the Tef-induced increase in the *I*_Na(P)_ amplitude activated by the upright isosceles-triangular ramp at either upsloping or downsloping limb, and (f) it mildly inhibited the amplitude of *I*_K(DR)_, *I*_K(erg)_, or *I*_h_, and (g) in HL-1 cardiomyocytes, and CBN was effective at depressing *I*_Na_ as well as at decreasing the τ_inact(S)_ value of the current. The experimental results enable us to reflect that CBN-mediated change in the amplitude and gating kinetics of ionic currents presented herein tends to be upstream of its action either on cytosolic NOD1/Nf-κB activation [2,5,31] or on the activity of antioxidant enzymes [14], and that it could conceivably participate in the adjustments on different functional activities in electrically excitable cells (e.g., GH_3_ or HL-1 cells) occurring in vivo.

It is important to mention that the effect of CBN on the time course of *I*_Na_ inactivation is continuously changing over time. Cell exposure to CBN tends to raise the rate of current inactivation during rapid membrane depolarization, enabling us to suggest that such molecule has the propensity to reach the blocking site only when the Na_V_ channel resides in the open or inactivated state. As such, aside from its effective inhibition of peak *I*_Na_ amplitude, the CBN molecule may primarily interact on the activation and/or inactivation process, presumably resulting in the adjustments on both the magnitude and gating kinetics of the current, although the detailed underlying mechanism of its action on *I*_Na_ still remains to be resolved. Indeed, according to quantitative estimate from the heuristic binding scheme stated above, the *K*_D_ value required for CBN-perturbed inactivation time course of *I*_Na_ (i.e., τ_inact(S)_) seen in GH_3_ cells was yielded to be 3.15 µM, a value which is actually close to the IC_50_ value needed for its inhibition of late *I*_Na_. Additionally, since the block of peak *I*_Na_ caused by CBN was noticed to consist of tonic and use-dependent components, it is likely that the intrinsic inactivation kinetics of the current could be altered in its presence.

In this study, the presence of CBN was unable to modify the steady-state activation curve (i.e., *G*_Na_ versus membrane potential relationship) of peak *I*_Na_ identified in GH_3_ cells. However, the steady-state inactivation curve of peak *I*_Na_ during its exposure (10 µM) was noticed to shift toward a hyperpolarized potential by about 9 mV; however, there is void of changes in the gating charge of the curve. Consequently, the sensitivity of nonvoltage-clamped excitable cells (e.g., neurons or endocrine cells) to this agent would be assumed to rely on different confounding variables, which include the pre-existing level of resting membrane potential, the pattern of action potential firing, the CBN concentration achieved, or that occurring in any combinations [20].

In the present study, the voltage-dependent hysteresis of *I*_Na(P)_ during exposure to Tef was observed by the long-lasting upright isosceles-triangular ramp voltage command with a ramp speed of 0.04 mV/ms, although such hysteresis was barely observed in the control (i.e., Tef was not present). The peak *I*_Na(P)_ activated at the upsloping limb of such triangular ramp was noted to be placed at around −0 mV, while that at the downsloping limb was markedly shifted to −60 mV. Tef-induced *I*_Na(P)_ existing detected in GH_3_ cells was observed to undergo hysteresis in its voltage dependence. It is likely that such *I*_Na(P)_ during the exposure to Tef could intrinsically possess “memory” of previous events (or dynamic voltage dependence of *I*_Na(P)_) or that mode shift occurs with respect to the voltage sensitivity of gating charge movement, which relies on the previous state (or conformation) of the Na_V_-channel [32,33]. Such unique hysteretic behavior would play substantial role in influencing electrical behavior or sodium overload of excitable cells during exposure to such pyrethroid insecticides [23,27,28]. Moreover, the addition of CBN (10 µM), but not that of chlorotoxin (1 µM), still in the presence of Tef, was observed to be effective at attenuate Tef-induced *I*_Na(P)_ detected at both upsloping and downsloping limbs of the triangular ramp pulse.

The intestinal absorption and transportation of CBN has been previously reported [27,34]. This compound was noticed to be readily absorbed into blood stream and well distributed into various organs after intravenous or oral administration to rats [11,35,36,37]. Moreover, by use of liquid chromatography tandem mass spectrometry, the peak CBN level in rats following intravenous administration (5 mg/Kg) was previously measured to reach around 4.7 µg/mL (or 14 µM) [37,38]. This value is similar to effective IC_50_ required for its inhibition of peak *I*_Na_ presented herein; however, it is virtually higher than either that for block of late *I*_Na_ or the *K*_D_ value optimally estimated from the binding scheme. Therefore, it is likely that the CBN actions on ionic currents reported in GH_3_ or HL-1 cells are of therapeutic and pharmacological relevance, if similar in-vivo findings occur.

A recent study reported ineffectiveness of CBN in modifying *I*_Na_ in small dorsal root ganglion neurons [10]. Conversely, finding from our results disclosed that CBN could be effective at inhibiting the magnitude of *I*_Na_ inherently in GH_3_ cells, favorably at the late (pulse-end) component of the current, which coincided with the increased inactivation rate. This discrepancy is currently unknown; however, it could be due to the view that different Na_V_-channel isoforms or auxiliary α-subunits exist in various types of electrically excitable cells. It was noticed that the *SCN5A*-encoded or Na_V_1.5 current is robustly expressed in heart cells, while the Na_V_ isoforms have not been identified in pituitary GH_3_ cells; however, dorsal root ganglion neurons might express up to five isoforms, which are functionally active in these neurons, e.g., *SCN1A*-, *SCN8A*-, *SCN9A*-, *SCN10A*-, or *SCN11A*-encoded current (or Na_V_1.1, Na_V_1.6, Na_V_1.7, Na_V_1.8, or Na_V_1.9 current) [18,19]. Additional studies to delineate the effects of CBN on *I*_Na_ in different cell types that express different isoforms would be warranted.

Earlier and recent studies have demonstrated the ability of CBN to modify the amplitude of voltage-gated Ca^2+^ currents [10,16,17]. However, to some extent distinguishable from those results, the present observations hereby demonstrated that CBN-mediated inhibition of *I*_Na_ seen in GH_3_ cells could be further diminished by SSM, TTX, or Ran but not by Nim. Moreover, as cells were continually exposed to CBN, further addition of Tef, a type-I insecticide known to be an activator of *I*_Na_, could effectively reverse CBN-mediated reduction in *I*_Na_ in response to rapid depolarization. The IC_50_ values needed for CBN-mediated inhibition of peak and late *I*_Na_ observed in this study were estimated to be 14.7 and 2.8 µM, respectively, the values which were found to be virtually lower than the concentration (i.e., around 100 µM) used either for its block of Ca^2+^ currents in dorsal root ganglion neurons [10] or for the suppression of prolactin release from GH_3_ cells [16]. Additionally, according to the binding and unbinding scheme, the *K*_D_ value for CBN-mediated perturbation of *I*_Na_ inactivation was yielded to be 3.15 µM. Our study, therefore, reflects that in pituitary or heart cells, the presence of CBN does not appear to interact with plasmalemmal Ca^2+^ channels exclusively. It is nonetheless possible that aside from the inhibition of Ca^2+^ currents, CBN was efficacious at influencing the amplitude and gating kinetics of *I*_Na_, which at least a subset of Na_V_-channel isoforms constitutes. CBN-mediated perturbation of Ca^2+^ uptake previously observed in GH_3_ cells [16,17] could be partly, if not entirely, explained by its inhibitory actions on peak and late components of *I*_Na_. Of importance, to what extent CBN-mediated perturbations on the amplitude and gating of *I*_Na_ are connected with its alleviation of pain sensation as reported recently [6,8,10], remains to be further resolved.

## 4. Materials and Methods

### 4.1. Chemicals and Solutions Used in this Work

Columbianadin (CBN, zosmin, 2-butenoic acid, 1-(8,9-dihydro-2-oxo-2*H*-furo(2,3-h)-1-benzopyran-8-yl)-1-methylethyl ester, (S-(Z))-, 1-methyl-1-[(8S)-2-oxo-8,9-dihydro-2H-furo [2,3-h]chromen-8-yl]ethyl (2Z)-2-methylbut-2-enoate, 1-[(8*S*)-8,9-dihydro-2-oxo-2*H*-furo[2,3-h]-1-benzopyran-8-yl]-1-methylethyl-[(2*Z*)-2-methyl-2-butenoic acid]ester, C_19_H_20_O_5_, https://pubchem.ncbi.nlm.nih.gov/compound/Columbianadin) and nimodipine (Nim) were acquired from Cayman Chemical (Asia Bioscience, Taipei, Taiwan), ranolazine (Ran) was obtained from Tocris (Union Biomed, Taipei, Taiwan), dexmedetomidine (DEX) was obtained from Abbott Laboratories (Abbott Park, IL, USA), while ivabradine (IVA), norepinephrine, tefluthrin (Tef), tetraethylammonium chloride (TEA), and tetrodotoxin (TTX) were acquired from Sigma-Aldrich (Merck, Taipei, Taiwan). Sesamin (SSM) was a gift kindly provided by Dr. Ping-Chung Kuo, School of Pharmacy, National Cheng Kung University Medical College, Tainan, Taiwan, whereas chlorotoxin was gifted by Dr. Woei-Jer Chuang, Department of Biochemistry, National Cheng Kung University Medical College. CBN, DEX, IVA, Ran, SSM, or Tef was dissolved in DMSO as 20 mM stock solution, and it was thereafter diluted in extracellular solution to the final concentrations achieved. Stock solution of CBN was wrapped in aluminum foil to avoid photosensitivity [39,40]. Unless stated otherwise, culture media, horse serum, fetal bovine or calf serum, L-glutamine, penicillin-streptomycin, and trypsin/EDTA were acquired from HyClone^TM^ (Fisher Scientific, Taipei, Taiwan), while all other chemicals, such as aspartic acid, CsOH, CsCl, EGTA, and HEPES, were of laboratory grade and taken from standard sources.

The HEPES-buffered normal Tyrode’s solution used in this work had a composition, which contained (in mM): NaCl 136.5, KCl 5.4, CaCl_2_ 1.8, MgCl_2_ 0.53, glucose 5.5, and HEPES 5.5, and the solution pH was adjusted to 7.4 with NaOH. For measurements of *I*_Na_, *I*_K(DR)_, or *I*_h_, we bathed cells in Ca^2+^-free Tyrode’s solution in attempts to remove the contamination of Ca^2+^-activated K^+^ currents and voltage-gated Ca^2+^ currents. To record *I*_K(erg)_, cells were bathed in high-K^+^, Ca^2+^-free solution, which comprised (in mM) KCl 130, NaCl 10, MgCl_2_ 3, and HEPES 5, and the solution pH was adjusted to 7.4 with KOH. For investigations on either *I*_K(erg)_ or *I*_h_, we filled up the electrode with the internal solution (in mM): K-aspartate 130, KCl 20, KH_2_PO_4_ 1, MgCl_2_ 1, EGTA 0.1, Na_2_ATP 3, Na_2_GTP 0.1, and HEPES 5, adjusted to pH 7.2 with KOH, while for the recordings of *I*_Na_, K^+^ ions in the internal solution were substituted for Cs^+^ ions, and the solution pH was adjusted to 7.2 with CsOH. All solutions used in this study were prepared using demineralized water from Mill-Q water purification system (Merck, Taipei, Taiwan). On the day of experiments, we commonly filtered the bathing or backfilling solution and culture medium using an Acrodisc^®^ syringe filter with a 0.2-µm Supor^®^ nylon membrane (Bio-Check, New Taipei City, Taiwan).

### 4.2. Cell Preparations

GH_3_, a rat pituitary cell line derived from a pituitary tumor (BCRC-60015), was acquired from the Bioresources Collection and Research Center (Hsinchu, Taiwan) using procedure that we have previously reported [41]. Briefly, GH_3_ cells were grown as monolayer cultures in Ham’s F-12 medium supplemented with 15% horse serum (*v/v*), 2.5% fetal calf serum (*v/v*), and 2 mM L-glutamine. The HL-1 atrial cell line was derived from the AT-1 mouse atrial cardiomyocyte tumor lineage, which was originally acquired from Louisiana State University in New Orleans, LA, USA [42]. HL-1 cells were cultured in Claycomb medium (Sigma-Aldrich) supplemented with 10% fetal bovine serum (*v/v*), 100 U/mL penicillin, 100 mg/mL streptomycin, 0.1 mM norepinephrine, and 2 mM L-glutamine. GH_3_ or HL-1 cells were plated in 100-mm culture dishes (10^6^ cells/dish) and maintained at 37 °C in a humidified environment of 5% CO_2_/95% air. They underwent sub-cultured weekly, and fresh media were replenished every other day to remove nonadhering cells. Subcultures were obtained by trypsinization (0.025% trypsin solution (HyClone^TM^) containing 0.01% sodium *n*,*n*-diethyldithiocarbamate and EDTA). Under the experimental conditions studied, cells remained 80–90% viable for at least 2 weeks. Electrophysiological measurements were performed 5 or 6 days after the cells had been cultured (60–80% confluence).

### 4.3. Electrophysiological Measurements

On the day of the experiments, GH_3_ or HL-1 cells were harvested with 1% trypsin/EDTA solution, and a few drops of cell suspension was rapidly transferred to a home-made recording chamber that was mounted on the XY stage of an inverted DM-IL microscope (Leica; Major Products, New Taipei City, Taiwan). The cells studies were bathed at room temperature (20–25 °C) in HEPES-buffered normal Tyrode’s solution, the composition of which is elaborated above. The cells were then allowed to adhere to the bottom of the chamber. To pull the patch electrode from Kimax-51 capillaries (#34500 (outer diameter: 1.5–1.8 mm); Kimble; Dogger, New Taipei City, Taiwan), we utilized either a P-97 horizontal puller (Sutter; Taiwan Instruments, Tainan, Taiwan) or a PP-83 vertical puller (Narishige; Taiwan Instrument Co., Taipei, Taiwan), and their tips were fire-polished with MF-83 Microforge (Narishige). The electrodes used bore tip resistances between 3 and 5 MΩ when filled with different internal solutions. An electrode holder which was filled with a silver chloride-coated silver wire connected the patch electrode to the amplifier. During the recordings, the electrode used was maneuvered using an MX-4 manipulator (Narishige) and finely operated by an MHW-3 hydraulic micromanipulator (Narishige). We performed standard patch-clamp recordings under whole-cell configuration by use of either an RK-400 (Biologic, Echirolles, France) or an Axoclamp-2B (Molecular Devices; Advance Biotech, New Taipei City, Taiwan) amplifier [41]. Consistent with previous observations [43], the formation of a bleb of membrane lipids in the electrode tip during microscopic observation of seal formation was also noticed. Giga-seals were generally formed in an all-or-nothing fashion and resulted in a dramatic improvement in signal-to-noise ratio. The junctional potentials, which develop at the electrode tip when the composition of the internal solution differed from that in the bath, were nulled before the start of each giga-seal formation, and such junction potentials corrected whole-cell data. Tested compounds were applied by perfusion (2–3 mL/min) or added to the bath to obtain the final concentration achieved.

### 4.4. Curve-Fitting Procedures and Statistical Analyses

The pertinent parameters for linear or nonlinear current fitting were appropriately acquired by either nonlinear (e.g., Boltzmann and Hill equation, or single- and two-exponential function) or linear fitting routine, in which the Solver add-in (i.e., the generalized reduced gradient method of iteration) bundled with Excel 2016 (Microsoft, Redmond, WA, USA) or 64-bit OriginPro 2016 (OriginLab; Schmidt Scientific, Kaohsiung, Taiwan) was undertaken [44]. The data acquired in this study are presented as the mean ± standard error of the mean (SEM), with sample sizes (*n*) representing the number of cells (e.g., GH_3_ or HL-1 cells) where the experimental data were taken. Student’s *t*-test (paired or unpaired) was initially employed for the statistical analyses; however, as the differences among different groups needed to be further evaluated, we further performed either analysis of variance (ANOVA)-1 or ANOVA-2 with or without repeated measures, which were followed by Duncan’s post hoc test. To evaluate the sum of squared residuals (SSR) as a function of the IC_50_ value for the inhibitory action of CBN on peak or late *I*_Na_, the 95% confidence interval was estimated using Fisher’s *F* distribution (i.e., FINV function embedded in Microsoft Excel) [44], since the Excel FINV function can calculate the inverse of the right-tailed F probability distribution for a supplied probability. A *p*-value of <0.05 was considered to indicate statistical different, unless stated otherwise.

### 4.5. Data Recordings

The signals consisting of voltage and current tracings were monitored and digitally stored at 10 kHz in an Acer SPIN-5 touchscreen laptop computer (SP51-52N-WE; Acer, Taipei, Taiwan), and data acquisition in conjunction with Digidata 1440A (Molecular Devices) was operated by pCLAMP 10.7 software (Molecular Devices). In some of the experiments, wu employed a PowerLab data acquisition via LabChart 7.0 program to digitize signals reliably (AD Instruments; KYS Technology, Tainan, Taiwan). To minimize background noise, the current signals were low-pass filtered at 2 kHz with FL-4 four-pole Bessel filter (Dagan, Minneapolis, MN, USA). Through digital-to-analog conversion, the pCLAMP-generated waveforms (i.e., either rectangular or ramp voltage-clamp command potentials) were specifically designed and applied to the examined cells for the determination of current-voltage (*I-V*) relationship and study either steady-state activation or inactivation curve of the current specified (e.g., *I*_Na_). As high-frequency stimuli were needed, we employed an Astro-Med Grass S85X dual output pulse stimulator (Grass, West Warwick, RI, USA).

### 4.6. Data Analyses

To establish percentage inhibition of CBN on the peak and late components of *I*_Na_, cells were bathed in Ca^2+^-free, Tyrode’s solution, the examined cell was voltage-clamped at −80 mV, and the 40-ms depolarizing pulse from −80 to −10 mV was thereafter delivered to evoke *I*_Na_. Current amplitudes during the application of varying CBN concentrations were compared with those measured after subsequent addition of TTX (1 µM). The concentration-dependent relation of CBN on the inhibition of *I*_Na_ measured at the start or end of the 40-ms depolarizing pulse from −80 to −10 mV was appropriately fitted to the Hill equation (i.e., the multiparameter logistic equation). That is,

(1) $$\mathrm{Percentage}\;\mathrm{inhibition}=\frac{{\left[\mathrm{CBN}\right]}^{n_{H}}\times E_{\max}}{{\left[\mathrm{CBN}\right]}^{n_{H}}+{\mathrm{IC}}_{50}^{n_{H}}},$$

where [CBN] is the CBN concentration; IC_50_ or n_H_ is the CBN concentration for 50% *I*_Na_ inhibition or the Hill coefficient, respectively; and E_max_ is the CBN-induced maximal inhibition of peak or late *I*_Na_ (i.e., TTX-sensitive current).

The inhibitory effect of CBN on *I*_Na_ is thought to ascribe from a state-dependent blocker that binds predominantly to the open state of the Na channel [22]. Based on this simplifying assumption, a minimal kinetic scheme was thereafter given as follows:

(2) $$C\;\overset{\alpha }{\underset{\beta }{⇄}}\;O\;\overset{k_{+1}{}^{*}\cdot \left[\mathrm{CBN}\right]}{\underset{k_{-1}\;}{⇄}}O\cdot \mathrm{CBN}$$

or,

(3) $$\frac{dC}{dt}=O\times \beta -C\times \alpha$$

$$\frac{dO}{dt}=C\times \alpha +O\cdot CBN\times k_{-1}-O\times \beta -O\times k_{+1}^{*}\cdot \left[\mathrm{CBN}\right]\;\frac{d\left(O\cdot CBN\right)}{dt}=O\times k_{+1}^{*}\cdot \left[CBN\right]-O\cdot CBN\times k_{-1},$$

where [CBN] is the CBN concentration applied and α or β is the voltage-gated rate constant for the opening or closing of the Na_V_ channel elicited by rapid step depolarization, respectively. *k*_+1_* or *k*_−1_ indicates the forward (i.e., on or blocking) or backward (i.e., off or unblocking) rate constant of CBN, respectively, whereas C, O, or O·CBN in each term represents the closed (resting), open, or open-blocked state, respectively.

The value of *k*_+1_* or *k*_−1_ was calculated on the basis of the time constant of the slow component in *I*_Na_ inactivation (τ_inact(S)_) in response to 40-ms depolarizing pulse achieved during the exposure to varying CBN concentrations. By virtue of the above-described binding scheme, these rate constants could be allowed to be optimized using the following equation:

$$\frac{1}{\tau_{inact\left(S\right)}}=\left[CBN\right]\times k_{+1}^{*}+k_{-1},$$

where *k*_+1_* or *k*_−1_ can be derived from the slope or from the *y*-axis intercept at [CBN] = 0 of the linear regression, which interpolates the reciprocal time constants (i.e., 1/τ_inact(S)_) versus the CBN concentration used, and [CBN] is the CBN concentration. A measure of the dissociation constant (*K*_D_) equal to the *k*_−1_ value divided by the value of [CBN]·*k*_+1_* can thereafter reliably be yielded.

The steady-state activation or inactivation curve of peak *I*_Na_ with or without the CBN addition was established and plotted against either membrane or conditioning potential, respectively, and the sigmoidal curve of the current was least-squares fitted by the Boltzmann equation (or the Fermi-Dirac distribution) [33]:

$$I\;\left(\mathrm{or}\;G_{Na}\right)=\frac{I_{max}\left(or\;G_{Na\left(\max\right)}\right)}{1+e^{\frac{\pm \left(V-V_{1/2}\right)\times q\times F}{RT}}},$$

where *I*_max_ or *G*_Na(max)_, respectively, indicates the maximal amplitude or conductance of *I*_Na_ in the absence and presence of CBN (10 µM); *V* is the membrane or conditioning potential in mV; V_1/2_ is the membrane potential at which half-maximal activation or inactivation of *I*_Na_ is achieved; *q* is the apparent activation or inactivation gating charge; *F* is Faraday’s constant; *R* is the universal gas constant; and *T* is the absolute temperature.

## Data Availability

The original data is available upon reasonable request to the corresponding author.

## Abbreviations

ANOVA	analysis of variance
CBN	columbianadin
DEX	dexmedetomidine
*G*_Na_	Na^+^-current conductance
I-V	current versus voltage
IC_50_	half-maximal inhibitory concentration
*I*_h_	hyperpolarization-activated cation current
*I*_K(erg)_	*erg*-mediated K^+^ current
*I*_Na_	voltage-gated Na^+^ current
IVA	ivabradine
*K*_D_	dissociation constant
Na_V_ channel	voltage-gated Na^+^ channel
Nim	nimodipine
Ran	ranolazine
SEM	standard error of mean
SSM	sesamin
SSR	sum of the squared residuals
τ_inact(S)_	time constant for the slow component of *I*_Na_ inactivation
Tef	tefluthrin
TEA	tetraethylammonium chloride
TTX	tetrodotoxin
